# *N*-Cyclo­hexyl-*N*-(prop-2-en-1-yl)benzene­sulfonamide

**DOI:** 10.1107/S1600536809048077

**Published:** 2009-11-18

**Authors:** Islam Ullah Khan, Zeeshan Haider, Muhammad Zia-ur-Rehman, Muhammad Nadeem Arshad, Muhammad Shafiq

**Affiliations:** a Department of Chemistry, Government College University, Lahore 54000, Pakistan; b Applied Chemistry Research Centre, PCSIR Laboratories Complex, Ferozpure Road, Lahore 54600, Pakistan

## Abstract

The title compound, C_15_H_21_NO_2_S, synthesized by *N*-alkyl­ation of cyclo­hexyl­amine benzene­sulfonamide with allyl iodide, is of inter­est as a precursor to biologically active sulfur-containing heterocyclic compounds. The cyclo­hexane ring is in a chair form and its mean plane makes a dihedral angle of 53.84 (12)° with the phenyl ring.

## Related literature

For the synthesis of related mol­ecules, see: Arshad *et al.* (2009 ▶); Zia-ur-Rehman *et al.* (2009 ▶). For biological applications of sulfonamides, see: Connor (1998 ▶); Berredjem *et al.* (2000 ▶); Lee & Lee (2002 ▶); Xiao & Timberlake (2000 ▶). For a related structure, see: Khan *et al.* (2009 ▶). For bond-length data, see: Allen *et al.* (1987 ▶).
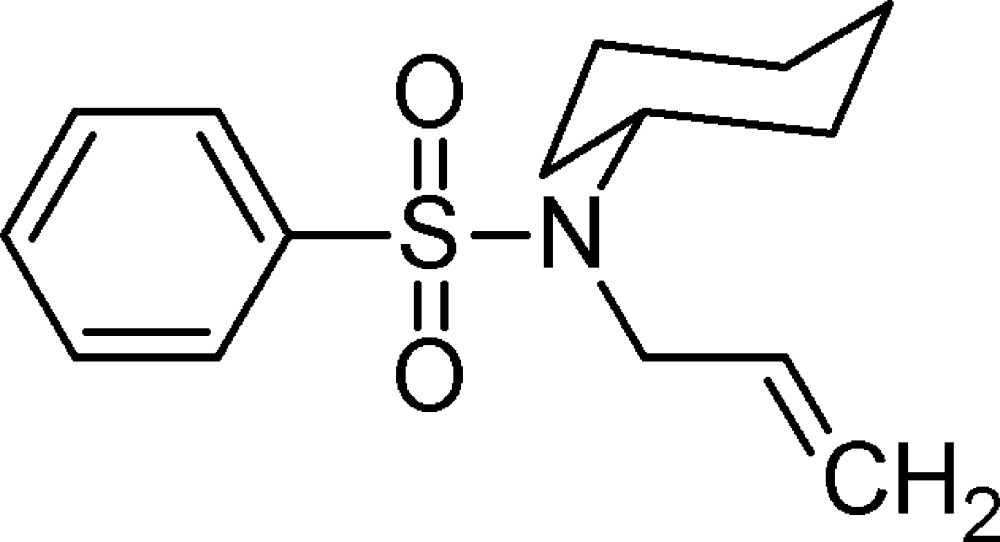


## Experimental

### 

#### Crystal data

C_15_H_21_NO_2_S
*M* *_r_* = 279.39
Monoclinic, 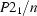

*a* = 8.4911 (5) Å
*b* = 11.4176 (6) Å
*c* = 15.6274 (10) Å
β = 94.188 (3)°
*V* = 1511.00 (15) Å^3^
*Z* = 4
Mo *K*α radiation
μ = 0.21 mm^−1^
*T* = 296 K
0.38 × 0.18 × 0.12 mm

#### Data collection

Bruker APEXII CCD area-detector diffractometer
Absorption correction: multi-scan (**SADABS**; Sheldrick, 1996 ▶) *T*_min_ = 0.924, *T*_max_ = 0.975
16559 measured reflections
3746 independent reflections
2179 reflections with *I* > 2σ(*I*)
*R*_int_ = 0.047

#### Refinement

*R*[*F* ^2^ > 2σ(*F* ^2^)] = 0.052
*wR*(*F* ^2^) = 0.144
*S* = 1.02
3746 reflections
172 parameters
H-atom parameters constrained
Δρ_max_ = 0.43 e Å^−3^
Δρ_min_ = −0.34 e Å^−3^

### 

Data collection: *APEX2* (Bruker, 2007 ▶); cell refinement: *SAINT* (Bruker, 2007 ▶); data reduction: *SAINT*; program(s) used to solve structure: *SHELXS97* (Sheldrick, 2008 ▶); program(s) used to refine structure: *SHELXL97* (Sheldrick, 2008 ▶); molecular graphics: *PLATON* (Spek, 2009 ▶) and *Mercury* (Macrae *et al.*, 2006 ▶); software used to prepare material for publication: *WinGX* (Farrugia, 1999 ▶) and *PLATON*.

## Supplementary Material

Crystal structure: contains datablocks I, global. DOI: 10.1107/S1600536809048077/is2487sup1.cif

Structure factors: contains datablocks I. DOI: 10.1107/S1600536809048077/is2487Isup2.hkl

Additional supplementary materials:  crystallographic information; 3D view; checkCIF report

## Figures and Tables

**Fig. 1.**
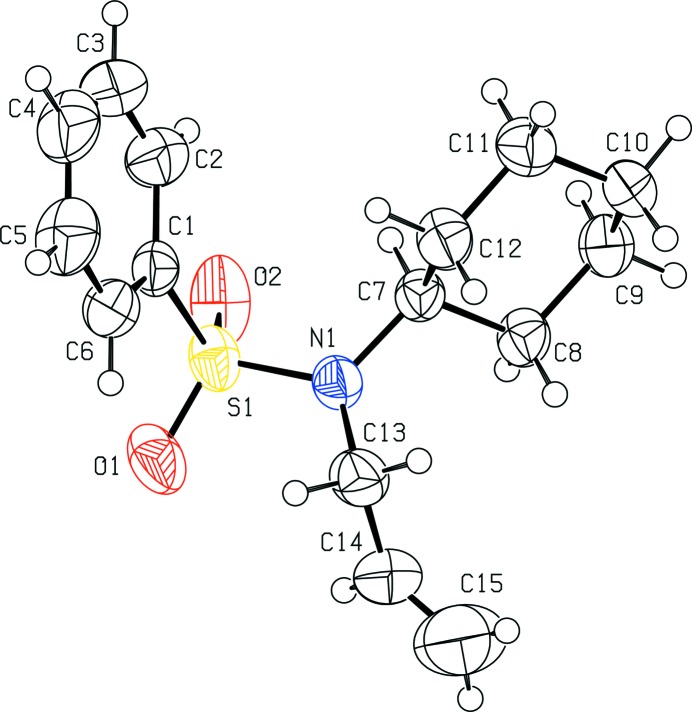
The molecular structure of the title compound, with displacement ellipsoids at the 50% probability level.

## supplementary crystallographic information

### Comment

Sulfonamides are familiar in the literature for their anti-malarial, anti-convulsant and anti-hypertensive (Connor, 1998; Xiao & Timberlake, 2000; Berredjem *et al.*, 2000; Lee & Lee, 2002) activities. As a part of our ongoing research program regarding the synthesis of sulfur containing heterocyclic compounds (Arshad *et al.*, 2009; Zia-ur-Rehman *et al.*, 2009; Khan *et al.*, 2009), we herein report the crystal structure of the title compound (Fig. 1).

In the title molecule, bond lengths and bond angles are within the normal ranges (Allen *et al.*, 1987). In the crystal structure, the phenyl ring is essentially planar while the cyclohexane ring is in a chair form. No significant hydrogen bond interactions are observed in the title molecule. The dihedral angle between the phenyl and cyclohexane rings is 53.84 (12)°.

### Experimental

A mixture of *N*-cyclohexylbenzene sulfonamide (1.0 g, 0.43 mmol), sodium hydride (0.21 g, 0.88 mmol) and *N*, *N*-dimethylformamide (10.0 ml) was stirred at room temperature for half an hour followed by addition of allyl iodide (0.144 g, 0.86 mmol). Stirring was continued further for a period of three hours and the contents were poured over crushed ice. Precipitated product was isolated, washed and crystallized from methanol.

### Refinement

All hydrogen atoms were identified in the difference map. However, they were fixed in ideal positions and treated as riding on their parent atoms. The following distances were used: C_methyl_—H = 0.98 Å and C_aromatic_—H = 0.95 Å. *U*_iso_(H) was set to 1.2*U*_eq_(C) or 1.5*U*_eq_(C_methyl_).

### Crystal data

**Table:**

C_15_H_21_NO_2_S	*F*(000) = 600
*M**_r_* = 279.39	*D*_x_ = 1.228 Mg m^−^^3^
Monoclinic, *P*2_1_/*n*	Mo *K*α radiation, λ = 0.71073 Å
Hall symbol: -P 2yn	Cell parameters from 3312 reflections
*a* = 8.4911 (5) Å	θ = 2.2–21.3°
*b* = 11.4176 (6) Å	µ = 0.21 mm^−^^1^
*c* = 15.6274 (10) Å	*T* = 296 K
β = 94.188 (3)°	Needles, light yellow
*V* = 1511.00 (15) Å^3^	0.38 × 0.18 × 0.12 mm
*Z* = 4	

### Data collection

**Table:**

Bruker APEXII CCD area-detector diffractometer	3746 independent reflections
Radiation source: fine-focus sealed tube	2179 reflections with *I* > 2σ(*I*)
graphite	*R*_int_ = 0.047
φ and ω scans	θ_max_ = 28.3°, θ_min_ = 2.2°
Absorption correction: multi-scan (*SADABS*; Sheldrick, 1996)	*h* = −11→11
*T*_min_ = 0.924, *T*_max_ = 0.975	*k* = −15→15
16559 measured reflections	*l* = −19→20

### Refinement

**Table:**

Refinement on *F*^2^	Primary atom site location: structure-invariant direct methods
Least-squares matrix: full	Secondary atom site location: difference Fourier map
*R*[*F*^2^ > 2σ(*F*^2^)] = 0.052	Hydrogen site location: inferred from neighbouring sites
*wR*(*F*^2^) = 0.144	H-atom parameters constrained
*S* = 1.01	*w* = 1/[σ^2^(*F*_o_^2^) + (0.0594*P*)^2^ + 0.3938*P*] where *P* = (*F*_o_^2^ + 2*F*_c_^2^)/3
3746 reflections	(Δ/σ)_max_ < 0.001
172 parameters	Δρ_max_ = 0.43 e Å^−^^3^
0 restraints	Δρ_min_ = −0.34 e Å^−^^3^

### Special details

**Table:**

Geometry. All e.s.d.'s (except the e.s.d. in the dihedral angle between two l.s. planes) are estimated using the full covariance matrix. The cell e.s.d.'s are taken into account individually in the estimation of e.s.d.'s in distances, angles and torsion angles; correlations between e.s.d.'s in cell parameters are only used when they are defined by crystal symmetry. An approximate (isotropic) treatment of cell e.s.d.'s is used for estimating e.s.d.'s involving l.s. planes.
Refinement. Refinement of *F*^2^ against ALL reflections. The weighted *R*-factor *wR* and goodness of fit *S* are based on *F*^2^, conventional *R*-factors *R* are based on *F*, with *F* set to zero for negative *F*^2^. The threshold expression of *F*^2^ > σ(*F*^2^) is used only for calculating *R*-factors(gt) *etc*. and is not relevant to the choice of reflections for refinement. *R*-factors based on *F*^2^ are statistically about twice as large as those based on *F*, and *R*- factors based on ALL data will be even larger.

### Fractional atomic coordinates and isotropic or equivalent isotropic displacement parameters (Å^2^)

**Table:**

	*x*	*y*	*z*	*U*_iso_*/*U*_eq_	
S1	0.27668 (7)	0.31209 (5)	0.25071 (4)	0.0519 (2)	
O1	0.3713 (2)	0.21435 (17)	0.27943 (11)	0.0744 (6)	
O2	0.3425 (2)	0.42730 (16)	0.25495 (14)	0.0776 (6)	
N1	0.1228 (2)	0.31154 (15)	0.30554 (12)	0.0460 (5)	
C1	0.2123 (2)	0.28696 (18)	0.14300 (14)	0.0438 (5)	
C2	0.1843 (3)	0.3808 (2)	0.08789 (19)	0.0714 (8)	
H2	0.2024	0.4570	0.1073	0.086*	
C3	0.1297 (4)	0.3605 (3)	0.0047 (2)	0.0942 (11)	
H3	0.1097	0.4233	−0.0324	0.113*	
C4	0.1044 (4)	0.2497 (4)	−0.02421 (19)	0.0881 (10)	
H4	0.0692	0.2370	−0.0812	0.106*	
C5	0.1303 (3)	0.1558 (3)	0.03010 (18)	0.0744 (8)	
H5	0.1110	0.0801	0.0101	0.089*	
C6	0.1849 (3)	0.1740 (2)	0.11428 (16)	0.0534 (6)	
H6	0.2032	0.1108	0.1513	0.064*	
C7	0.0098 (2)	0.41024 (18)	0.29605 (14)	0.0416 (5)	
H7	0.0635	0.4740	0.2679	0.050*	
C8	−0.0307 (3)	0.45575 (19)	0.38291 (14)	0.0485 (6)	
H8A	0.0654	0.4776	0.4164	0.058*	
H8B	−0.0819	0.3943	0.4136	0.058*	
C9	−0.1402 (3)	0.5618 (2)	0.37271 (16)	0.0584 (7)	
H9A	−0.1702	0.5861	0.4287	0.070*	
H9B	−0.0841	0.6263	0.3482	0.070*	
C10	−0.2872 (3)	0.5350 (2)	0.31574 (16)	0.0573 (6)	
H10A	−0.3497	0.4769	0.3433	0.069*	
H10B	−0.3503	0.6055	0.3078	0.069*	
C11	−0.2460 (3)	0.4895 (2)	0.22941 (16)	0.0621 (7)	
H11A	−0.1928	0.5504	0.1993	0.074*	
H11B	−0.3421	0.4689	0.1953	0.074*	
C12	−0.1393 (3)	0.3822 (2)	0.23980 (15)	0.0539 (6)	
H12A	−0.1959	0.3190	0.2655	0.065*	
H12B	−0.1109	0.3563	0.1838	0.065*	
C13	0.0737 (3)	0.20179 (19)	0.34549 (16)	0.0566 (6)	
H13A	0.1046	0.1358	0.3114	0.068*	
H13B	−0.0404	0.2007	0.3464	0.068*	
C14	0.1475 (4)	0.1896 (2)	0.4359 (2)	0.0795 (9)	
H14	0.2546	0.2069	0.4439	0.095*	
C15	0.0842 (6)	0.1601 (3)	0.4985 (3)	0.1242 (14)	
H15A	−0.0228	0.1417	0.4943	0.149*	
H15B	0.1420	0.1557	0.5513	0.149*	

### Atomic displacement parameters (Å^2^)

**Table:**

	*U*^11^	*U*^22^	*U*^33^	*U*^12^	*U*^13^	*U*^23^
S1	0.0389 (3)	0.0632 (4)	0.0529 (4)	0.0041 (3)	−0.0012 (3)	−0.0167 (3)
O1	0.0604 (11)	0.1025 (14)	0.0580 (12)	0.0354 (10)	−0.0124 (9)	−0.0179 (10)
O2	0.0527 (10)	0.0808 (13)	0.1011 (15)	−0.0240 (9)	0.0184 (10)	−0.0401 (11)
N1	0.0466 (11)	0.0490 (10)	0.0423 (11)	0.0079 (8)	0.0035 (9)	−0.0034 (8)
C1	0.0376 (11)	0.0515 (12)	0.0429 (13)	0.0029 (9)	0.0074 (10)	−0.0026 (10)
C2	0.090 (2)	0.0625 (16)	0.0642 (19)	0.0097 (14)	0.0200 (16)	0.0112 (14)
C3	0.114 (3)	0.112 (3)	0.058 (2)	0.044 (2)	0.0161 (19)	0.026 (2)
C4	0.072 (2)	0.149 (3)	0.0426 (17)	0.027 (2)	−0.0041 (14)	−0.005 (2)
C5	0.0732 (19)	0.093 (2)	0.0562 (18)	−0.0054 (16)	0.0002 (15)	−0.0247 (16)
C6	0.0576 (15)	0.0552 (14)	0.0473 (14)	0.0014 (11)	0.0037 (12)	−0.0037 (11)
C7	0.0413 (12)	0.0455 (11)	0.0380 (12)	0.0012 (9)	0.0026 (10)	−0.0027 (9)
C8	0.0505 (13)	0.0538 (13)	0.0409 (13)	0.0021 (10)	0.0010 (11)	−0.0096 (10)
C9	0.0706 (17)	0.0520 (13)	0.0534 (15)	0.0092 (12)	0.0098 (13)	−0.0102 (11)
C10	0.0553 (15)	0.0609 (14)	0.0561 (16)	0.0170 (12)	0.0065 (12)	0.0024 (12)
C11	0.0565 (15)	0.0786 (17)	0.0496 (16)	0.0154 (13)	−0.0053 (12)	−0.0003 (13)
C12	0.0515 (14)	0.0666 (15)	0.0424 (14)	0.0077 (11)	−0.0056 (11)	−0.0133 (11)
C13	0.0663 (16)	0.0484 (13)	0.0549 (16)	0.0073 (11)	0.0034 (13)	−0.0028 (11)
C14	0.097 (2)	0.0728 (18)	0.070 (2)	0.0197 (16)	0.0169 (18)	0.0238 (16)
C15	0.164 (4)	0.111 (3)	0.097 (3)	0.014 (3)	0.009 (3)	0.025 (2)

### Geometric parameters (Å, °)

**Table:**

S1—O1	1.4283 (18)	C8—H8A	0.9700
S1—O2	1.4289 (17)	C8—H8B	0.9700
S1—N1	1.6136 (19)	C9—C10	1.511 (4)
S1—C1	1.755 (2)	C9—H9A	0.9700
N1—C13	1.474 (3)	C9—H9B	0.9700
N1—C7	1.481 (2)	C10—C11	1.510 (3)
C1—C6	1.380 (3)	C10—H10A	0.9700
C1—C2	1.385 (3)	C10—H10B	0.9700
C2—C3	1.368 (4)	C11—C12	1.525 (3)
C2—H2	0.9300	C11—H11A	0.9700
C3—C4	1.355 (5)	C11—H11B	0.9700
C3—H3	0.9300	C12—H12A	0.9700
C4—C5	1.375 (4)	C12—H12B	0.9700
C4—H4	0.9300	C13—C14	1.509 (4)
C5—C6	1.378 (4)	C13—H13A	0.9700
C5—H5	0.9300	C13—H13B	0.9700
C6—H6	0.9300	C14—C15	1.198 (4)
C7—C8	1.516 (3)	C14—H14	0.9300
C7—C12	1.522 (3)	C15—H15A	0.9300
C7—H7	0.9800	C15—H15B	0.9300
C8—C9	1.528 (3)		
			
O1—S1—O2	119.70 (12)	H8A—C8—H8B	108.1
O1—S1—N1	106.79 (11)	C10—C9—C8	111.75 (18)
O2—S1—N1	107.95 (10)	C10—C9—H9A	109.3
O1—S1—C1	107.60 (10)	C8—C9—H9A	109.3
O2—S1—C1	106.76 (12)	C10—C9—H9B	109.3
N1—S1—C1	107.52 (10)	C8—C9—H9B	109.3
C13—N1—C7	119.21 (17)	H9A—C9—H9B	107.9
C13—N1—S1	119.58 (14)	C11—C10—C9	111.2 (2)
C7—N1—S1	119.19 (14)	C11—C10—H10A	109.4
C6—C1—C2	120.3 (2)	C9—C10—H10A	109.4
C6—C1—S1	119.87 (17)	C11—C10—H10B	109.4
C2—C1—S1	119.82 (19)	C9—C10—H10B	109.4
C3—C2—C1	119.4 (3)	H10A—C10—H10B	108.0
C3—C2—H2	120.3	C10—C11—C12	110.8 (2)
C1—C2—H2	120.3	C10—C11—H11A	109.5
C4—C3—C2	120.7 (3)	C12—C11—H11A	109.5
C4—C3—H3	119.7	C10—C11—H11B	109.5
C2—C3—H3	119.7	C12—C11—H11B	109.5
C3—C4—C5	120.5 (3)	H11A—C11—H11B	108.1
C3—C4—H4	119.8	C7—C12—C11	110.82 (19)
C5—C4—H4	119.8	C7—C12—H12A	109.5
C4—C5—C6	120.0 (3)	C11—C12—H12A	109.5
C4—C5—H5	120.0	C7—C12—H12B	109.5
C6—C5—H5	120.0	C11—C12—H12B	109.5
C5—C6—C1	119.2 (2)	H12A—C12—H12B	108.1
C5—C6—H6	120.4	N1—C13—C14	111.3 (2)
C1—C6—H6	120.4	N1—C13—H13A	109.4
N1—C7—C8	111.04 (17)	C14—C13—H13A	109.4
N1—C7—C12	113.81 (17)	N1—C13—H13B	109.4
C8—C7—C12	110.80 (18)	C14—C13—H13B	109.4
N1—C7—H7	106.9	H13A—C13—H13B	108.0
C8—C7—H7	106.9	C15—C14—C13	127.5 (4)
C12—C7—H7	106.9	C15—C14—H14	116.2
C7—C8—C9	110.76 (19)	C13—C14—H14	116.2
C7—C8—H8A	109.5	C14—C15—H15A	120.0
C9—C8—H8A	109.5	C14—C15—H15B	120.0
C7—C8—H8B	109.5	H15A—C15—H15B	120.0
C9—C8—H8B	109.5		
			
O1—S1—N1—C13	−22.4 (2)	C2—C1—C6—C5	0.3 (4)
O2—S1—N1—C13	−152.36 (18)	S1—C1—C6—C5	178.25 (19)
C1—S1—N1—C13	92.80 (18)	C13—N1—C7—C8	64.3 (2)
O1—S1—N1—C7	173.87 (15)	S1—N1—C7—C8	−131.93 (17)
O2—S1—N1—C7	43.96 (19)	C13—N1—C7—C12	−61.5 (3)
C1—S1—N1—C7	−70.88 (17)	S1—N1—C7—C12	102.2 (2)
O1—S1—C1—C6	32.0 (2)	N1—C7—C8—C9	176.87 (18)
O2—S1—C1—C6	161.62 (18)	C12—C7—C8—C9	−55.6 (2)
N1—S1—C1—C6	−82.75 (19)	C7—C8—C9—C10	55.1 (3)
O1—S1—C1—C2	−150.1 (2)	C8—C9—C10—C11	−55.4 (3)
O2—S1—C1—C2	−20.4 (2)	C9—C10—C11—C12	56.1 (3)
N1—S1—C1—C2	95.2 (2)	N1—C7—C12—C11	−177.19 (19)
C6—C1—C2—C3	−0.2 (4)	C8—C7—C12—C11	56.8 (3)
S1—C1—C2—C3	−178.1 (2)	C10—C11—C12—C7	−56.9 (3)
C1—C2—C3—C4	−0.6 (5)	C7—N1—C13—C14	−104.9 (2)
C2—C3—C4—C5	1.3 (5)	S1—N1—C13—C14	91.5 (2)
C3—C4—C5—C6	−1.1 (5)	N1—C13—C14—C15	134.8 (4)
C4—C5—C6—C1	0.3 (4)		
